# Somalier: rapid relatedness estimation for cancer and germline studies using efficient genome sketches

**DOI:** 10.1186/s13073-020-00761-2

**Published:** 2020-07-14

**Authors:** Brent S. Pedersen, Preetida J. Bhetariya, Joe Brown, Stephanie N. Kravitz, Gabor Marth, Randy L. Jensen, Mary P. Bronner, Hunter R. Underhill, Aaron R. Quinlan

**Affiliations:** 1 Department of Human Genetics, University of Utah, 15 S 2030 E, Salt Lake City, UT 84112 USA; 2 Base2 Genomics, LLC, Salt Lake City, UT 84105 USA; 3 Department of Neurosurgery, Radiation Oncology and Oncological Sciences, Huntsman Cancer Institute, University of Utah, 5th Floor CNC, 175 North Medical Drive, Salt Lake City, UT 84132 USA; 4 Department of Pathology, Huntsman Cancer Institute, University of Utah, 15 S 2030 E, Salt Lake City, UT 84112 USA; 5 Division of Medical Genetics, Department of Pediatrics, Department of Radiology, University of Utah, 15 S 2030 E, Salt Lake City, UT 84112 USA; 6 Department of Biomedical Informatics, University of Utah, 421 Wakara Way #140, Salt Lake City, UT 84108 USA

## Abstract

**Background:**

When interpreting sequencing data from multiple spatial or longitudinal biopsies, detecting sample mix-ups is essential, yet more difficult than in studies of germline variation. In most genomic studies of tumors, genetic variation is detected through pairwise comparisons of the tumor and a matched normal tissue from the sample donor. In many cases, only somatic variants are reported, which hinders the use of existing tools that detect sample swaps solely based on genotypes of inherited variants. To address this problem, we have developed Somalier, a tool that operates directly on alignments and does not require jointly called germline variants. Instead, Somalier extracts a small sketch of informative genetic variation for each sample. Sketches from hundreds of germline or somatic samples can then be compared in under a second, making Somalier a useful tool for measuring relatedness in large cohorts. Somalier produces both text output and an interactive visual report that facilitates the detection and correction of sample swaps using multiple relatedness metrics.

**Results:**

We introduce the tool and demonstrate its utility on a cohort of five glioma samples each with a normal, tumor, and cell-free DNA sample. Applying Somalier to high-coverage sequence data from the 1000 Genomes Project also identifies several related samples. We also demonstrate that it can distinguish pairs of whole-genome and RNA-seq samples from the same individuals in the Genotype-Tissue Expression (GTEx) project.

**Conclusions:**

Somalier is a tool that can rapidly evaluate relatedness from sequencing data. It can be applied to diverse sequencing data types and genome builds and is available under an MIT license at github.com/brentp/somalier.

## Background

DNA sequencing data from matched tumor-normal pairs are critical for the detection of somatic variation in cancer studies. However, a sample swap leads to a dramatic increase in the apparent number of somatic variants, confounds the genetic analysis of the tumor, and the probability of such a mix-up increases with the size of the study cohort. The correction for sample mix-ups, possibly a swap with another sample in the same study, requires a thorough evaluation of the coefficient of relationship (henceforth “relatedness”) among the entire set of samples, as measured by the similarity of their genotypes at polymorphic loci. This is not possible directly on the somatic mutation predictions because somatic variants are typically detected from comparisons of tumor-normal pairs, and often, only somatic (not germline) variants are reported [1]. Therefore, resolution of the sample swap would require the researcher to jointly call germline variants with a tool like GATK [2] and then use methods such as peddy [3] or KING [4] to calculate relatedness across the entire set of samples. Joint variant calling is time and resource intensive, especially when all that is needed to resolve the sample swap is an accurate calculation of relatedness among the samples. After experiencing this inconvenience in our own research, we developed Somalier to quickly and accurately compute relatedness by extracting “sketches” of variant information directly from alignments (BAM or CRAM) or from variant call format (VCF) [5] files including genomic VCFs (GVCF). Somalier extracts a sketch for each sample and the sketches are then compared to evaluate all possible pairwise relationships among the samples. This setup mitigates the “N+1 problem” by allowing users to add new sketches as needed and efficiently compare them to an existing set of background samples. The text and visual output facilitates the detection and correction of sample swaps, even in cases where there is severe loss of heterozygosity. It can be used on any organism across diverse sequencing data types and, given a set of carefully selected sites, across genome builds. We show that Somalier produces similar kinship estimates to KING [4] in much less time and that it produces reliable measures across tissue types and when comparing DNA samples, RNA-seq samples, and DNA to RNA-seq samples.

## Implementation

### Selecting and extracting informative variant sites

We have previously shown that using as few as 5000 carefully chosen polymorphic loci is sufficient for relatedness estimation and that this subset of informative loci yields more accurate estimates than using all available variants [3]. A similar site-selection strategy is also used in Conpair to estimate contamination [6]. In Somalier, we utilize the observation that the optimal sites for detecting relatedness are high-quality, unlinked sites with a population allele frequency of around 0.5. A balanced allele frequency maximizes the probability that any 2 unrelated samples will differ. We distribute a set of informative sites to be queried by Somalier, though users may also create their own sites files tailored to their application. The sites are high-frequency single-nucleotide variants selected from gnomAD [7] exomes that exclude segmental duplication and low-complexity regions [8]. We also distribute a set of sites limited to exons that are frequently (> 10 reads in at least 40% of samples) expressed in GTeX for use in cohorts with RNA-seq data. To minimize genotyping error, variants with nearby insertions or deletions are excluded. In addition, we have excluded sites that are cytosines in the reference so that the tool can be used on bisulfite seq data, for example, to check the correspondence between bisulfite sequencing and RNA-seq data. The Somalier repository includes the code to create a set of sites for different organisms given a population VCF and a set of optional exclude regions. We distribute a default set of matched sites for both the GRCh37 and GRCh38 builds of the human reference genome. This allows a user to extract sites from a sample aligned to GRCh37 using our GRCh37 sites file and compare that sketch to a sketch created from a sample aligned to GRCh38 by extracting the sites in our GRCh38 file. This is convenient as labs move from GRCh37 to GRCh38 and future genome builds. The sites files include informative variants on the X and Y chromosomes so that Somalier can also estimate a sample’s sex from the genotypes. However, only autosomal sites are used to estimate relatedness. With the default sites files, Somalier inspects 17,766 total sites (these are distributed with the Somalier software), all of which are chosen to be in coding sequence so that they are applicable to genome, exome, and RNA-seq datasets.

In order to quickly extract data from polymorphic sites into a genome sketch, Somalier uses the BAM or CRAM index to query each file at each of the informative sites. Alignments with a mapping quality of at least 1 that are not duplicates, supplementary, or failing quality control (according to the SAM flag) are used. Each passing alignment is evaluated at the requested position and the base in the alignment at that position is checked against the given reference and alternate for the query variant. This check considers the CIGAR operation [9] at that base which indicates insertions, deletions, and other events within the read. This is faster than a traditional sequence alignment “pileup” as it looks at each read only once and interrogates only the exact position in question. If a VCF (or BCF or GVCF) is provided instead of an alignment file, Somalier will extract the depth information for each sample for requested sites that are present in the VCF. The sketches extracted from a VCF are indistinguishable from those extracted from alignment files. In order to support single-sample VCFs, which do not contain calls where the individual is homozygous for the reference allele, the user may indicate that missing variants should be assumed to be homozygous for the reference allele. This also facilitates comparing multiple tumor-normal VCFs where many sites will not be shared (however, in those cases, it is preferable to extract the sketch from the alignment files rather than from the VCF).

Somalier tallies reference and alternate counts for each site. Once all sites are collected, it writes a binary file containing the sample name and the allele counts collected at each of the inspected sites. For the set of sites distributed from the Somalier repository, a sketch file requires ~ 200 KB of space on disk or in memory. This sketch format and the speed of parsing and comparing sketch files are key strengths of Somalier. For example, since Somalier can complete a full analysis of 2504 sketches from the 1000 Genomes high-coverage whole-genome samples (Michael Zody, personal communication) in under 20 s, users can keep a pool of sample sketches to test against and check incoming samples against all previously sketched samples.

### Comparing sketches

**Fig. 1:**
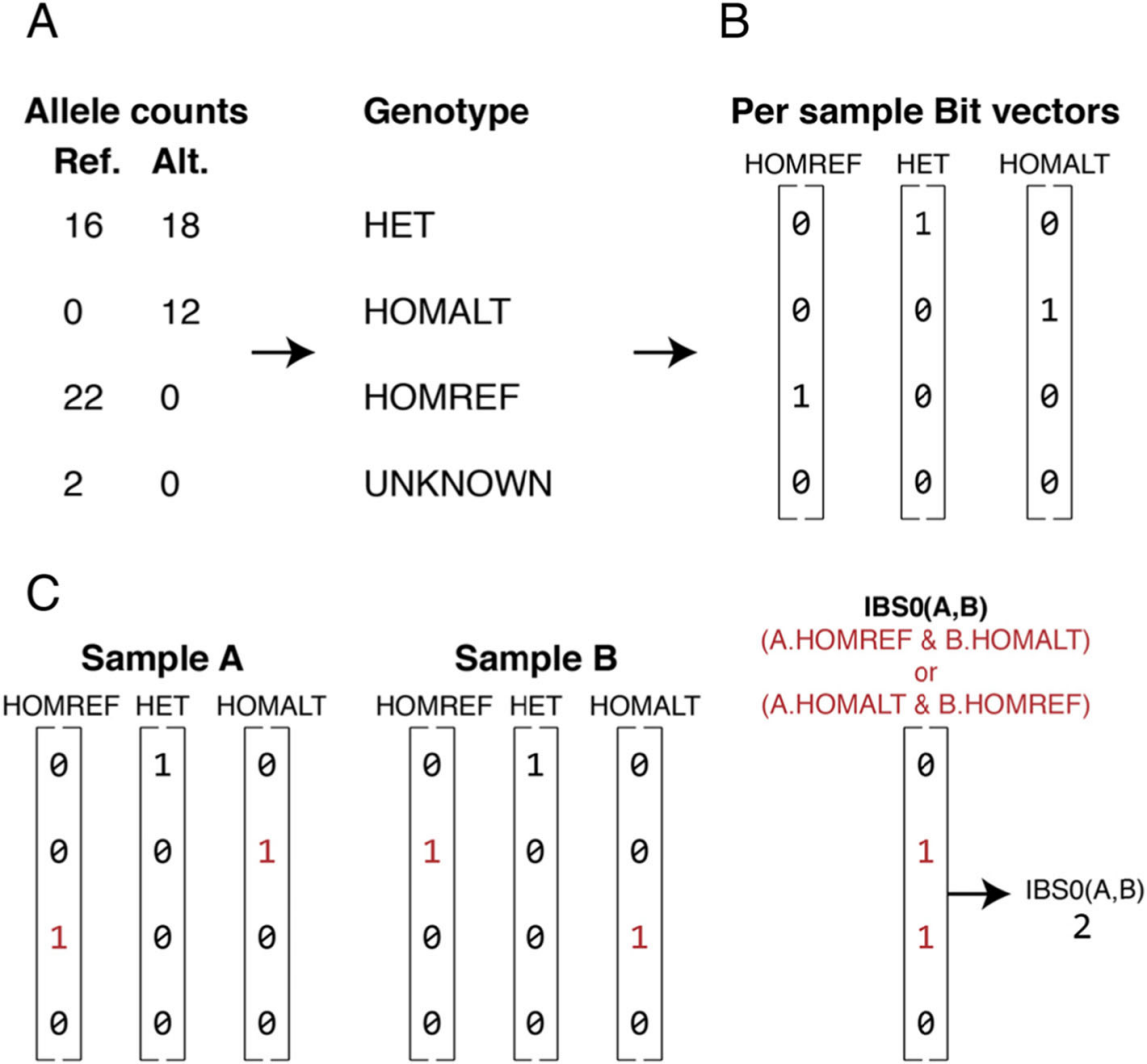
Comparing genotype sketches to compute relatedness measures for pairs of samples. **a** Observed counts for the reference (Ref.) and alternate (Alt.) allele at each of the tested 17,766 loci are converted into genotypes (see main text for details) to create a “sketch” for each sample. **b** The genotypes for each sample are then converted into three bit vectors: one for homozygous reference (HOMREF) genotypes, one for heterozygous (HET) genotypes, and one for homozygous alternate (HOMALT) genotypes. The length of each vector is the total number of autosomal variants in the sketch (i.e., 17,384) divided by 64, and the value for each bit is set to 1 if the sample has the particular genotype at the given variant site. For example, four variant sites are shown in **b** and the hypothetical individual has a homozygous alternate genotype for the second variant (the corresponding bit is set to 1), but is not homozygous for the alternate allele at the other three variant sites (the corresponding bits are set to 0). **c** The bit vectors for a pair of samples can be easily compared to calculate relatedness measures such as identity-by-state zero (IBS0, where zero alleles are shared between two samples) through efficient, bitwise operations on the bit arrays for the relevant genotypes

Thousands of sample sketches can be read into memory per second and compared. In order to calculate relatedness, Somalier converts the reference and alternate allele counts stored for each sample at each site into a genotype. The genotype is determined to be unknown if the depth is less than the user-specified value (default of 7), homozygous reference if the allele balance (i.e., alt-count/[ref-count + alt-count]) is less than 0.02, heterozygous if the allele balance is between 0.2 and 0.8, homozygous alternate if the allele balance is above 0.98, and unknown otherwise (Fig. 1a). A flag can amend these rules such that missing sites (with depth of 0) are treated as homozygous reference, rather than unknown. While simple, this heuristic genotyping works well in practice and is extremely fast, because Somalier looks only at single-nucleotide variants in non-repetitive regions of the genome. As the sample is processed, Somalier also collects information on depth, mean allele-balance, number of reference, heterozygous, and homozygous alternate calls for each sample, along with similar stats for the X and Y chromosomes. These data are used to calculate per-sample quality control metrics. In order to measure relatedness, the data collected for each sample is converted into a data structure consisting of *hom_ref*, *het*, and *hom_alt* bit vectors (Fig. 1b). The bit vectors consist of 64 bit integers, enabling Somalier to store 64 variants per integer. There are 17,384 autosomal sites in the default sites file used by Somalier, consuming only 6519 bytes per sample (17,384/64 bits * 3 bit-vectors/sample * 8 bits/byte). With this data layout, Somalier can represent all 2504 samples from the 1000 Genomes Project in under 17 megabytes of memory. This simple data structure also facilitates rapid pairwise comparisons (Fig. 1c); for example, we can compute IBS0 (that is, “identity by-state 0” or sites where zero alleles are shared between two samples *A* and *B*) with the following logic which evaluates 64 sites in parallel:

$$ \left(\mathtt{A}.\mathtt{\hom}\_\mathtt{ref}\ \mathtt{and}\ \mathtt{B}.\mathtt{\hom}\_\mathtt{alt}\right)\ \mathtt{or}\ \left(\mathtt{B}.\mathtt{\hom}\_\mathtt{ref}\ \mathtt{and}\ \mathtt{A}.\mathtt{\hom}\_\mathtt{alt}\right) $$

We repeat this for each of the 272 (17,384 autosomal sites/64 sites per entry) entries in the array to assess all of the genome-wide sites for each pair of samples. In fact, we do not need the sites, just the count of sites that are IBS0. Therefore, we use the *popcount* (i.e., the count of bits that are set to TRUE) hardware instruction to count the total number of bits where the expression is non-zero in order to get the total count of IBS0 sites between the 2 samples. In addition to IBS0, we calculate counts of IBS2 where both samples have the same genotype, shared heterozygotes (both are heterozygotes), shared homozygous alternates, and heterozygous sites for each sample. All of the operations are extremely fast as it does not require code branching via, for example, conditional logic; instead, the calculations are all conducted with bitwise operations.

Once those metrics are calculated, the relatedness between sample *i* and sample *j* is calculated as:

$$ \left(\mathtt{shared}\hbox{-} \mathtt{hets}\left(\mathtt{i},\mathtt{j}\right)\hbox{-} \mathtt{2}\ast \mathtt{ibs}\mathtt{0}\left(\mathtt{i},\mathtt{j}\right)\right)/\mathtt{\min}\ \left(\mathtt{hets}\left(\mathtt{i}\right),\mathtt{hets}\left(\mathtt{j}\right)\right) $$

where *hets* is the count of heterozygote calls per sample out of the assayed sites. This metric is derived by Manichaikul et al. [4]. In addition, the *homozygous concordance* rate is reported as:

$$ \left(\mathtt{shared}\hbox{-} \mathtt{homozygous}\hbox{-} \mathtt{alts}\left(\mathtt{i},\mathtt{j}\right)\hbox{-} \mathtt{2}\ast \mathtt{ibs}\mathtt{0}\left(\mathtt{i},\mathtt{j}\right)\right)/\mathtt{\min}\ \left(\mathtt{homozygous}\hbox{-} \mathtt{alts}\left(\mathtt{i}\right),\mathtt{homozygous}\hbox{-} \mathtt{alts}\left(\mathtt{j}\right)\right) $$

This measure is similar to the one described in HYSYS [10] except that the HYSYS measure is simply:

$$ \Big(\mathtt{shared}\hbox{-} \mathtt{homozygous}\hbox{-} \mathtt{alts}\left(\mathtt{i},\mathtt{j}\right)\hbox{-} /\mathtt{\min}\ \left(\mathtt{homozygous}\hbox{-} \mathtt{alts}\left(\mathtt{i}\right),\mathtt{homozygous}\hbox{-} \mathtt{alts}\left(\mathtt{j}\right)\right) $$

Our formulation has the benefit that it matches the scale and interpretation of the relatedness estimate; unrelated individuals will have a concordance of around 0, whereas in HYSYS they will have a value around 0.5. This is a useful relatedness metric when severe loss of heterozygosity removes many heterozygous calls from the tumor sample making the traditional relatedness calculation inaccurate.

If a pedigree file is given, Wright’s method of path coefficients [11] is used to calculate the expected relatedness. These values can then be compared to the relatedness observed from the genotypes. For somatic samples, the user can also specify a “groups” file where sample identifiers appearing on the same line are expected to be identical; for example, three biopsies from each of two individuals would appear as three comma-separated sample identifiers on two separate lines.

Finally, the output is reported both as text and as an interactive HTML page. When using the webpage, the user can toggle which relatedness metrics (IBS0, IBS2, relatedness, homozygous concordance, shared heterozygotes, shared homozygous alternates) to plot for the X and Y coordinates and, if expected groups were given (e.g., tumor-normal pairs) on the command-line, points are colored according to their expected relatedness. This setup means that points of similar colors should cluster together. In addition, Somalier plots the per-sample output in a separate plot with selectable axes; this functionality allows one to evaluate predicted vs. reported sex and depth across samples.

Somalier requires htslib (https://htslib.org). It is written in the Nim programming language (https://nim-lang.org) which compiles to C and also utilizes our *hts-nim* [12] library. It is distributed as a static binary, and the source code is available at https://github.com/brentp/somalier under an academic license.

## Results

### Glioma patients with 3 samples

**Fig. 2:**
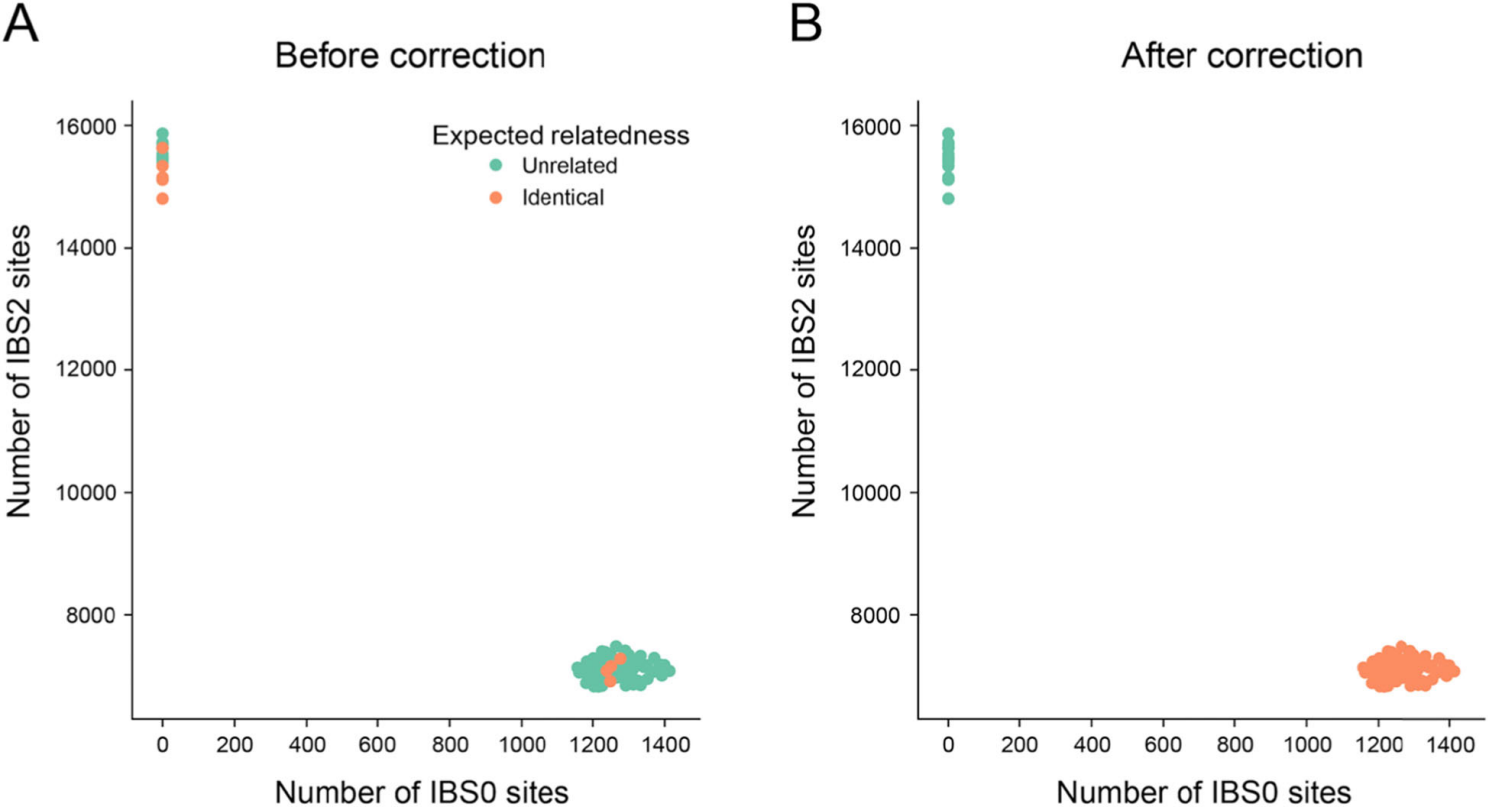
Glioma samples before and after correction. **a** A comparison of the IBS0 (number of sites where 1 sample is homozygous reference and another is homozygous alternate) and IBS2 (count of sites where samples have the same genotype) metric for 15 samples. Each point is a pair of samples. Points are positioned by the values calculated from the alignment files (observed relatedness) and colored by whether they are expected to be identical (expected relatedness), as indicated from the command line. In this case, sample swaps are visible as orange points that cluster with green points, and vice versa. The user is able to hover on each point to see the sample pair involved and to change the X and Y axes to any of the metrics calculated by Somalier. **b** An updated version of the plot in **a** after the sample identities have been corrected (per the information provided by **a**) in the manifest after re-running Somalier

We ran Somalier on BAM alignment files from five individuals, each with three assays: a normal sample, a glioma tumor sample, and cell-free DNA, for a total of 15 samples [13]. The extraction step, which creates the genome sketch and can be parallelized by sample, requires roughly three minutes per sample with a single CPU. Once extracted, the *relate* step, which computes the relatedness measures for each sample pair, requires less than 1 s. Somalier was able to clearly group samples using the default sites provided with the software (Fig. 2). Because the site selection is so strict, none of the sample pairs from the same individual had an IBS0 metric above 0, indicating that those sites are genotyped correctly. The user can specify expected groups of samples (e.g., from the same individual) with sample pairs expected to be identical colored as orange. With this layout that colors sample pairs by expected relatedness and positions them by observed relatedness (as computed from the genotypes estimated from the alignments), it is simple for the researcher to quickly spot problems. For example, Fig. 2a illustrates an obvious mix-up where samples expected to be unrelated have a high IBS2 and low IBS0. Since the plot is interactive, the user can then hover over points that appear out of place (in this example, the green points that cluster with the orange) to learn which samples are involved. After correcting the sample manifest based on this observation, and re-running the relatedness calculation, the updated plot shows that all samples cluster as expected given their relatedness (Fig. 2b).

### 1000 Genomes high-coverage samples

**Fig. 3:**
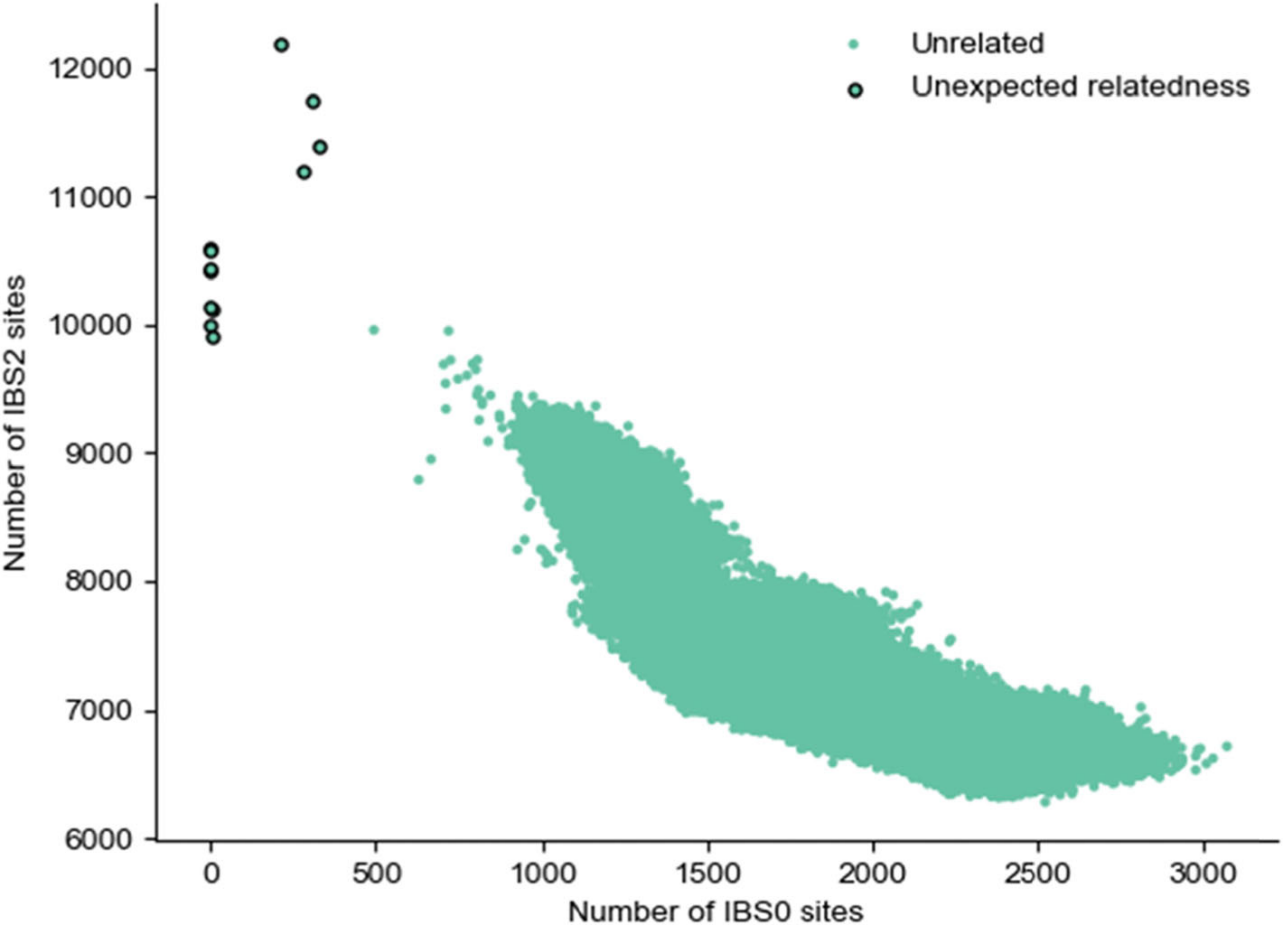
Relatedness plot for thousand genomes samples. Each dot represents a pair of samples. IBS0 on the *x*-axis is the number of sites where 1 sample is homozygous for the reference allele and the other is homozygous for the alternate allele. IBS2, on the *y*-axis, is the count of sites where a pair of samples were both homozygous or both heterozygous. Points with IBS0 of 0 are parent-child pairs. The 4 points with IBS0 > 0 and IBS0 < 450 are siblings. There are also several more distantly related sample pairs

In order to evaluate the scalability and accuracy of Somalier, we used the recently released high-coverage data from 2504 samples in the 1000 Genomes Project [14]. We extracted sites for all 2504 samples from the jointly called VCF. After extracting sketches, comparing each sample against all other samples (a total of 3,133,756 = 2504 * 2503 * 2 comparisons) required merely 6 s, following 1.1 s to parse the sketches and roughly 2 s to write the output. Although the 1000 Genomes Project provides a pedigree file, none of the samples included in the 2504 are indicated to be related by that file. However, using Somalier, we found 8 apparent parent-child pairs (NA19904-NA19913, NA20320-NA20321, NA20317-NA20318, NA20359-NA20362, NA20334-NA20335, HG03750-HG03754, NA20882-NA20900, NA20881-NA20900) 4 full-sibling pairs (HG02429-HG02479, NA19331-NA19334, HG03733-HG038899, HG03873-HG03998) and 3 second-degree relatives (NA19027-NA19042, NA19625-NA20274, NA21109-NA21135) (Fig. 3). These same sample pairs also have higher values (as expected) for homozygous concordance. In addition, there are several pairs of samples with a coefficient of relatedness between 0.1 and 0.2 that appear to be more distantly related. An earlier analysis on a different subset of the 1000 Genomes samples uncovered some of these same unreported relationships [15].

**Fig. 4:**
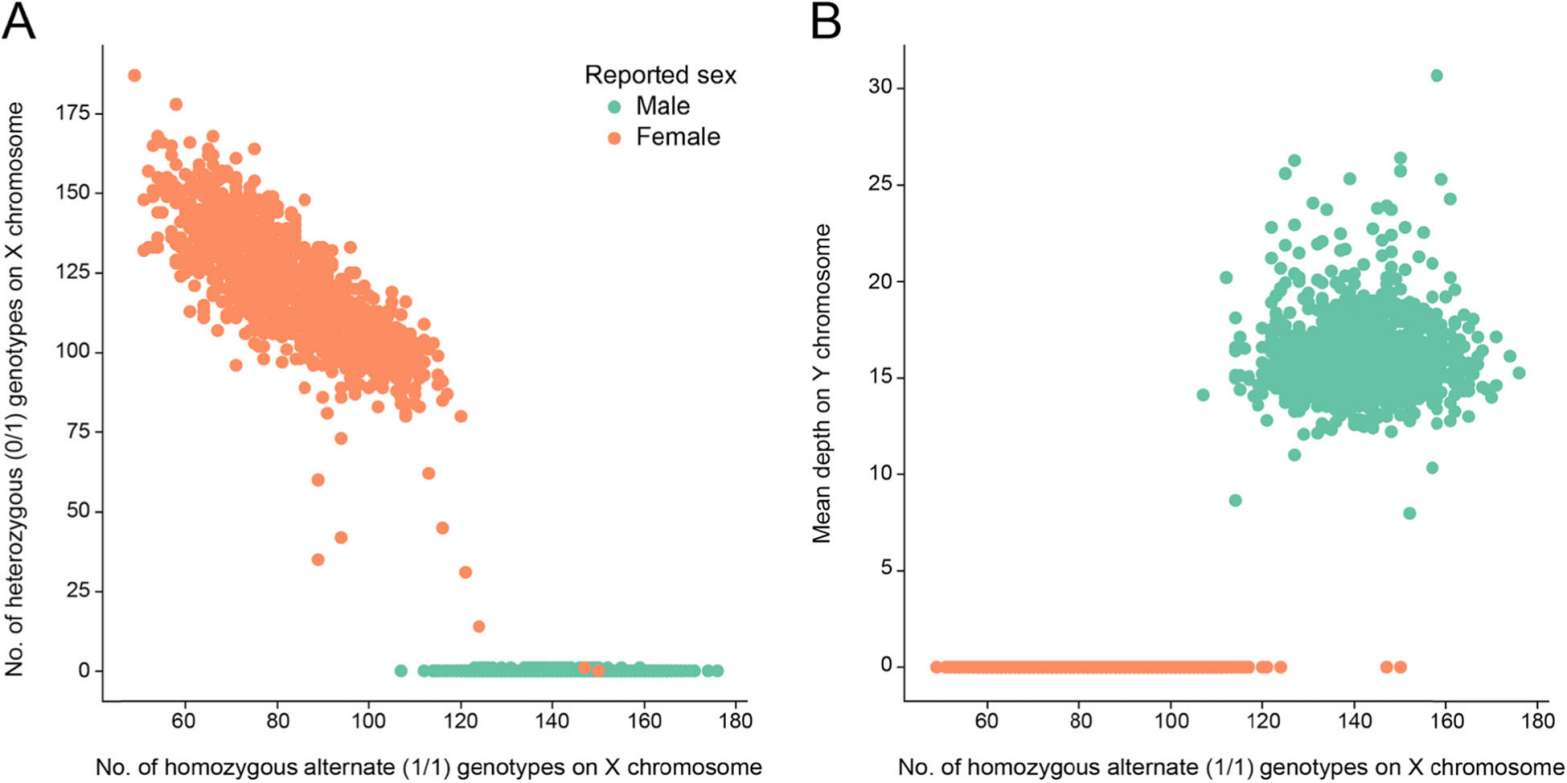
Sex quality control on thousand genomes samples. Each point is a sample colored as orange if the sample is indicated as female and green if it is indicated as male; all data is for the X chromosome. **a** The number of homozygous alternate sites on the *x*-axis and the number of heterozygous sites on the *y*-axis. Males and females separate with few exceptions. **b** The number of homozygous alternate sites on the *x*-axis compared to the mean depth on the Y chromosome. Males and females reported in the manifest separate perfectly, indicating that some females may have experienced a complete loss of the X chromosome

We also note that several samples indicated to be female in the manifest appear to have lost an X chromosome as they have lower depth and no heterozygous sites (Fig. 4a). However, they also lack coverage on the Y chromosome (Fig. 4); as such, we think that loss of X in these cell-line samples is more likely than a sample swap or manifest error. Finally, Somalier also provides other sample metrics including mean depth, counts of each genotype, mean allele balance, and others that are useful for sample quality control. The user can customize the visualization on the interactive web page by choosing which metrics to display on the X and Y axes.

### Comparison to KING

In order to evaluate the accuracy and speed of Somalier*,* we compared its performance to KING [4]. Since KING also has an extraction-like step, in converting VCF to PLINK’s [16] binary format, we partition the timing into distinct steps for data extraction (“extract”) and computation of relatedness (“relate”). We used KING version 2.2.4 and plink2 version 1.90p. We compared the speed and output of Somalier to that of KING on the 2504 thousand genomes, high-coverage VCF. Somalier is more flexible as it can be applied to VCF, BAMs, GVCFs, etc., but it is also faster both at extraction (which will only be done once) and at the relate step which can be repeated each time new samples are added (Table 1). Much of the speed improvement observed in Somalier comes from the sketch, which contains only a small subset of sites on the genome. Furthermore, kinship estimates from KING and Somalier are very similar (Additional file 1: Fig. S1).

**Table 1:** Speed comparison to KING. The extract step consists of conversion to a sketch for Somalier and of conversion to a plink binary bed file for KING. The relate step is the time spent measuring kinship between all pairs of samples. Times shown reflect the wall time required for completion

Step	Somalier (wall time)	KING/plink (wall time)
Extract	17 min 48 s	812 min 40 s
Relate	8 s	31 min 34 s

### Evaluation on GTeX RNA-seq and whole-genome seq

In order to show that Somalier can be used to find and verify sample identity across sequencing experiments and tissues, we applied it to a set of data from the GTeX project [17]. We utilized 216 samples with WGS, RNA-seq from skin (not sun exposed), and RNA-seq from blood. We expect each of these 648 assays to have a relatedness of 1 to the two other samples from the same GTeX individual. We used the per-exon, per-sample expression levels to create a set of sites that have relatively high allele frequency in gnomAD and are commonly expressed (> 10 reads in 40% of samples). These sites are distributed at the Somalier repository and include 16,469 autosomal sites and 794 sites on the X chromosome. We found that with a cutoff of relatedness of 0.5, which enforces that pairs of samples below this threshold are not from the same individual, we are able to correctly classify every sample pair (out of 209,628 pairs).

In order to show the specificity of Somalier with a smaller number of sites, we ran Somalier with 10, 20, 40, 100, 200, 400, 1000, 2000, 4000, 8000, and 16,000 of the original 16,469 autosomal sites and demonstrate that we are able to correctly classify all pairs with as few as 400 of the original 16,469 sites (Additional file 1: Fig. S2). If we instead require that unrelated samples have a calculated relatedness of less than 0.2 and related samples have greater than 0.8, then at least 1000 sites are required to reduce the false-positive rate (where unrelated samples are classified as related because they have a relatedness > 0.2) to under 0.05 (Additional file 2). Further, on inspection of the interactive Somalier plots (Additional file 3), it is clear that the false positives are driven by a few low-quality samples, each of which is involved in 647 pairs. If those were removed, the false-positive rates would drop.

## Discussion

We have introduced Somalier to efficiently detect sample swaps and mismatched samples in diverse DNA and RNA sequencing projects. On a set of 15 samples, we were able to detect and correct sample swaps using the text and HTML output from Somalier, which ran in less than a second. In addition, Somalier can be used to provide an accurate relatedness estimate using *homozygous concordance* even under severe loss-of-heterozygosity. We have designed it to measure relatedness very quickly despite assaying the alignments directly, and we have shown that using a carefully selected set of sites facilitates accurate separation of related from unrelated samples even on a small gene panel.

We have carefully selected the sites assayed by Somalier to minimize sequencing artifacts and variant calling errors. In addition, we distribute a set of sites for genome build GRCh37 that is compatible with genome build GRCh38. Because the sets are identical, we can compare samples aligned to either genome build. This becomes more important as research groups switch to GRCh38. In fact, in comparing the recently released high coverage 1000 Genomes samples (aligned to GRCh38) to the Simons Diversity Project samples [18] (aligned to GRCh37), we found several samples shared between these projects. To our knowledge, this has not been previously reported. These findings highlight the utility and novelty of Somalier, as it enables comparing across large cohorts.

Previous tools such as peddy provide similar functionality when a jointly called, germline VCF is provided. However, that is often not practical for cancer studies. In addition, HYSYS can detect sample swaps in cancer samples using homozygous concordance; however, it requires a custom text format which reports germline variants that have already been called across all samples. The sketch format used by Somalier is a simple binary format. We provide an example in the repository that demonstrates reading the data in a simple python script and performing ancestry estimation using principal components analysis. While Somalier can also utilize any number of VCF files as input, we expect that the simplicity and speed of using alignment files will make that the most frequent mode of use.

## Conclusions

We have introduced Somalier, a tool to rapidly evaluate relatedness from sequencing data in BAM, CRAM, or VCF formats. We show that it works across tissue types and to compare RNA-seq data to WGS. It is fast and simple to use and it simplifies analyses—such as comparison across cohorts and genomes builds—that were previously difficult or not feasible.

## Availability and requirements

Project name: Somalier

Project home page: https://github.com/brentp/somalier

Operating systems: Linux, OSX, Windows (a static binary is provided for Linux systems, users on other OSes can build the tool)

Programming language: Nim

License: MIT

## Supplementary information

**Additional file 1: Supplementary Fig. 1.** Comparison of KING estimate of kinship to that of Somalier. **Supplementary Fig. 2.** Evaluation of false-positive rate of Somalier as number of assayed sites is varied.

**Additional file 2.** Subset analysis for GTeX data.

**Additional file 3.** HTML output for GTeX analysis.

## Data Availability

The 1000 Genomes data [14] was downloaded from: http://ftp.1000genomes.ebi.ac.uk/vol1/ftp/data_collections/1000G_2504_high_coverage/working/20190425_NYGC_GATK/. The data used for the GTeX analyses described in this manuscript were analyzed on the Terra platform on April, 24, 2020. All procedures related to acquisition and use of data from glioma [13] patients were approved by the University of Utah Internal Review Board prior to study initiation (protocol #10924) and conform to the principles of the Helsinki Declaration. All glioma patients provided written informed consent. Due to the limitations imposed by the University of Utah Institutional Review Board (IRB), only a subset of the human sequencing data presented in this study can be made publicly available in a data repository. All glioma data was acquired between January 2016 and December 2016 under IRB #10924. All sequencing data acquired under IRB #10924 after January 1, 2015, and before August 23, 2017, cannot be shared in a public repository unless the patient is deceased. Bam files from three glioma patients are available in the NCBI Sequence Read Archive database under accession PRJNA641696 [13]. For those data that are unable to be shared via repository, please contact Ann Johnson (Ann.Johnson@hsc.utah.edu), Director of University of Utah IRB, to request access to the data.
