# Supplementary material for: Somalier: rapid relatedness estimation for cancer and germline studies using efficient genome sketches
Source: Genome Med. 2020 Jul 14;12:62. doi: 10.1186/s13073-020-00761-2 (PMC7362544; doi:10.1186/s13073-020-00761-2)
Supplement: Supplementary file 1 — Additional file 1: Supplementary Fig. 1. Comparison of KING estimate of kinship to that of Somalier. Supplementary Fig. 2. Evaluation of false-positive rate of Somalier as number of assayed sites is varied. [file 13073_2020_761_MOESM1_ESM.docx]

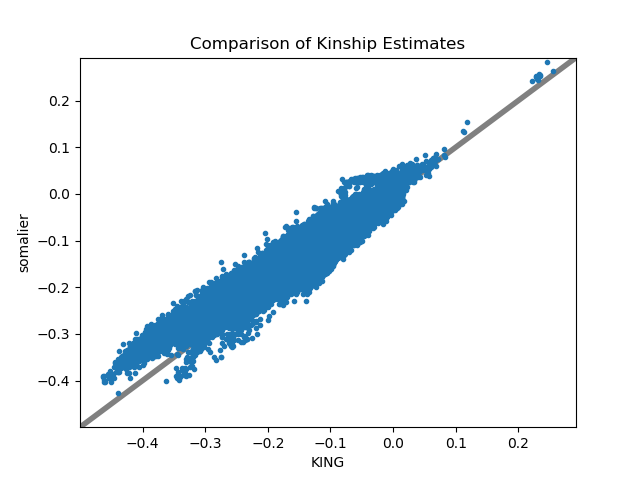


Additional File 1: Fig. S1. Comparison of KING estimate of kinship to that of *somalier*.


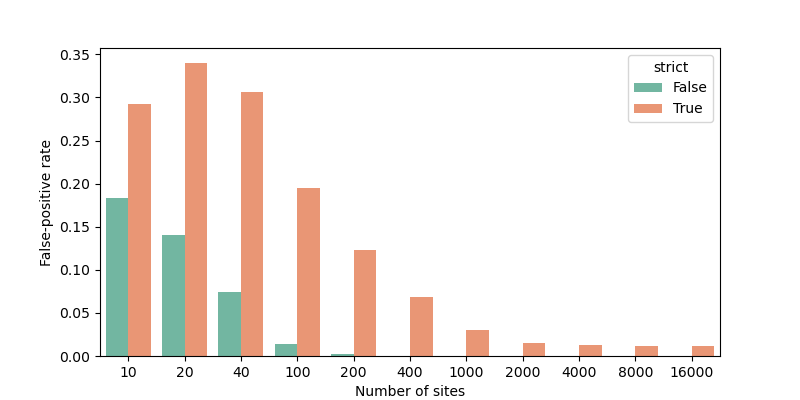


Additional File 2: Fig. S2. Evaluation of false-positive rate of *somalier* as number of assayed sites is varied. “Strict” mode requires that related samples have a relatedness > 0.8 and unrelated samples have a relatedness < 0.2. The non-strict mode requires that related samples have a relatedness value >= 0.5 and unrelated samples have a relatedness < 0.5. These are GTEx samples that are unrelated, but different tissues from the same individual are expected to have a relatedness value of 1.
