# Supplementary material for: Somalier: rapid relatedness estimation for cancer and germline studies using efficient genome sketches
Source: Genome Med. 2020 Jul 14;12:62. doi: 10.1186/s13073-020-00761-2 (PMC7362544; doi:10.1186/s13073-020-00761-2)
Supplement: Supplementary file 3 — Additional file 3. HTML output for GTeX analysis. [file 13073_2020_761_MOESM3_ESM.html]

somalier

##### Sample to Sample Relatedness

X Axis

Shared hets
Shared hom-alts
Homozygous concordance
Relatedness
IBS0
IBS2

Y Axis

Shared hets
Shared hom-alts
Homozygous concordance
Relatedness
IBS0
IBS2

##### Sample Depth Metrics

X Axis

Mean depth of genotyped sites
Mean depth of all sites
Mean allele balance
% reads with neither REF nor ALT
Number of 0/0 sites
Number of 0/1 sites
Number of 1/1 sites
Number of unknown sites
Number of known sites
Proportion sites with AB < 0.1 or AB > 0.9
Scaled mean depth on X
Number of 0/0 sites on X
Number of 0/1 sites on X
Number of 1/1 sites on X
Scaled mean depth on Y

Y Axis

Mean depth of genotyped sites
Mean depth of all sites
Mean allele balance
% reads with neither REF nor ALT
Number of 0/0 sites
Number of 0/1 sites
Number of 1/1 sites
Number of unknown sites
Number of known sites
Proportion sites with AB < 0.1 or AB > 0.9
Scaled mean depth on X
Number of 0/0 sites on X
Number of 0/1 sites on X
Number of 1/1 sites on X
Scaled mean depth on Y

Choose pre-set selection for sample plot:

- Sex QC: Number of 0/1 sites on X vs scaled mean depth on chrX
- Sex ploidy QC: Scaled mean depth on chrX vs. chrY
- General QC: number of sites with unknown genotype vs. proportion of hets with allele balance outside of 0.1 - 0.9
- Depth and allele QC: mean depth vs. mean het allele-balance
